# Bimatoprost Implant Biodegradation in the Phase 3, Randomized, 20-Month ARTEMIS Studies

**DOI:** 10.1089/jop.2022.0137

**Published:** 2023-01-25

**Authors:** Robert N. Weinreb, Jason Bacharach, Jacob W. Brubaker, Felipe A. Medeiros, Marina Bejanian, Paula Bernstein, Michael R. Robinson

**Affiliations:** ^1^ Hamilton Glaucoma Center and Viterbi Family Department of Ophthalmology, Shiley Eye Institute, University of California San Diego, La Jolla, California, USA.; ^2^ North Bay Eye Associates, Petaluma, California, USA.; ^3^ Sacramento Eye Consultants, Sacramento, California, USA.; ^4^ Duke Eye Center, Durham, North Carolina, USA.; ^5^ Allergan, an AbbVie company, Irvine, California, USA.; ^6^ Bernstein Biostatistics Consulting, LLC, Laguna Niguel, California, USA.

**Keywords:** biodegradable implant, drug delivery system, intraocular injection, prostaglandin analog, randomized clinical trial

## Abstract

**Purpose:**

To evaluate the time course of biodegradation of an intracameral, biodegradable, sustained-release bimatoprost implant that lowers intraocular pressure without the need for daily eye drops.

**Methods:**

In 2 identically designed, randomized, phase 3 clinical trials, adults with open-angle glaucoma or ocular hypertension and open iridocorneal angles inferiorly in the study eye were administered 10- or 15-μg bimatoprost implant (day 1 and weeks 16 and 32) or twice-daily topical timolol 0.5%. Implants were assessed on gonioscopy throughout the studies. Investigators reported whether implants were visible, estimated the size of visible implants relative to their initial size at implantation, and reported the implant location. Data for 10-μg implant placed on day 1 were pooled from both studies for analysis.

**Results:**

A total of 372 patients received the 10-μg bimatoprost implant. The degree of implant biodegradation at each follow-up time point was variable among patients. The implant frequently swelled during the initial phase of biodegradation from 6 to 28 weeks. Accelerated biodegradation occurred between 31 and 52 weeks, resulting in 82% of implants absent or ≤25% of initial size by 52 weeks. By month 20, 95% of implants had biodegraded to absent or ≤25% of initial size. The implant was predominantly located inferiorly in the iridocorneal angle.

**Conclusions:**

Bimatoprost implant biodegradation in phase 3 studies showed some degree of variability among patients. Clinically significant implant biodegradation was observed in the majority of patients by 12 months. Clinical studies are in progress to further understand implant biodegradation and the ideal timing for implant re-administration. ClinicalTrials.gov NCT02247804; ClinicalTrials.gov NCT02250651.

## Introduction

Bimatoprost implant 10 μg (Durysta; Allergan, an AbbVie company, North Chicago, IL) is an intracameral biodegradable implant used to lower intraocular pressure (IOP) in patients with open-angle glaucoma or ocular hypertension. The small, rod-shaped implant (diameter ∼200 μm and length ∼1.1 mm) contains 10 μg bimatoprost in an ophthalmic drug delivery system composed of poly-lactic acid and polylactic-*co*-glycolic polymers similar to those used in biodegradable sutures.^1^ The polymers in the implant have demonstrated safety and tolerability in ocular tissues.^2^ After the implant is administered intracamerally with a single-use, prefilled 28-gauge applicator system,^3^ the polymers are hydrolyzed and metabolized to carbon dioxide and water,^2^ and bimatoprost is released to lower IOP.

*In vitro* and *in vivo* data suggest that the implant releases bimatoprost for a period of approximately 3 to 4 months. Implants incubated in a buffered saline solution *in vitro* showed slow drug release in the first 2 weeks followed by a faster zero-order release profile until day 84, when the implants were nearly depleted of drug (Supplementary Fig. S1). *In vivo* in a beagle dog model, intraocular drug levels after bimatoprost implant administration were measurable through 80 days but were beneath the limit of quantitation at 4.2 months after implant administration.^1^ Also, in the phase 3 ARTEMIS 1 clinical study, drug concentrations in aqueous humor samples taken from 2 participants were below the limit of quantitation (<0.05 ng/mL) at 100 and 114 days, respectively, after their last implant administration.^1^

The effects of the bimatoprost implant on IOP typically last beyond the period of drug release and intraocular drug bioavailability. A single bimatoprost implant lowered IOP for up to 2 years in some patients in a phase 1/2 clinical study,^4^ and in both the ARTEMIS 1 and 2 randomized, masked, phase 3 studies, IOP was controlled in most patients without additional treatment at 1 year after a third bimatoprost implant administration.^1,5^ Furthermore, in a phase 3 clinical trial study extension, some patients continued to have controlled IOP and stable visual fields more than 3 years after receiving their last implant administration in the ARTEMIS study (Wirta et al, presented at the American Glaucoma Society 2022 Annual Meeting, Nashville, TN). Some patients in the study extension also had remnant implant remaining more than 3 years after receiving their last implant administration.

Remnant implant polymers also persist after the period of drug release from the implant.^1,4–6^ In the ARTEMIS 1 and 2 studies, patients received 3 administrations of bimatoprost implant 10 or 15 μg with a fixed 16-week dosing interval (day 1 and weeks 16 and 32). In each study, one or more residual implants remained visible in the iridocorneal angle on gonioscopy in over 80% of the patients at the month 20 study exit.^1,5^ The remnant implants were typically estimated to have decreased in size to less than or equal to 25% of their initial size.^1,5^

In both ARTEMIS studies, the 2 tested dose strengths of the bimatoprost implant effectively lowered IOP. However, the 10-μg bimatoprost implant had a more favorable benefit–risk profile, because corneal adverse events, thought to be related to the volume of implant material in the iridocorneal angle after multiple implant administrations at 16-week intervals, were more frequent with the larger 15-μg bimatoprost implant. Importantly, there were no reports of corneal edema, corneal endothelial cell loss, or corneal touch after a single administration of the 10-μg implant.^7^ Based on these findings, the current US Food and Drug Administration–approved use of the implant is limited to a single intracameral administration of bimatoprost implant 10 μg per eye, without retreatment.

Knowledge of the time course of bimatoprost implant biodegradation could be helpful in determining a safe interval for potential re-administration. Therefore, the objective of this study was to evaluate the degree of biodegradation of the bimatoprost implant over time in the phase 3 ARTEMIS studies. A secondary objective was to evaluate the morphology of the implant during biodegradation in a preclinical study.

## Methods

Two identically designed, randomized, multicenter, 20-month, phase 3 clinical trials (ARTEMIS 1 [ClinicalTrials.gov NCT02247804, reported by Medeiros et al^1^] and ARTEMIS 2 [ClinicalTrials.gov NCT02250651, reported by Bacharach et al^5^]) compared 10- and 15-μg bimatoprost implant with twice-daily topical timolol maleate 0.5% in patients with open-angle glaucoma or ocular hypertension. The studies were conducted in accordance with the Declaration of Helsinki and the Health Information Portability and Accountability Act, and institutional review board or ethics committee approval was obtained at each site. All participants provided written informed consent.

The patient eligibility criteria and methods for the ARTEMIS 1 and 2 studies have been reported in detail previously.^1,5^ Briefly, adults diagnosed with open-angle glaucoma or ocular hypertension in the study eye were enrolled. Key inclusion criteria included baseline IOP (after washout of any previous IOP-lowering medications) of 22–32 mmHg at hour 0 (8 am) and an open inferior iridocorneal angle (Shaffer grade of ≥3 on gonioscopy) in the study eye.

On day 1, enrolled patients were randomly assigned to treatment with 10-μg bimatoprost implant, 15-μg bimatoprost implant, or twice-daily topical timolol maleate 0.5%. Patients in the bimatoprost implant treatment groups received a 10- or 15-μg bimatoprost implant in the study eye on day 1 and weeks 16 and 32. The bimatoprost implant was administered intracamerally using sterile technique under standard aseptic conditions for intraocular procedures.^1^ A prefilled, single-use, 28-gauge sterile applicator was used for the administration.

**FIG. 1.**
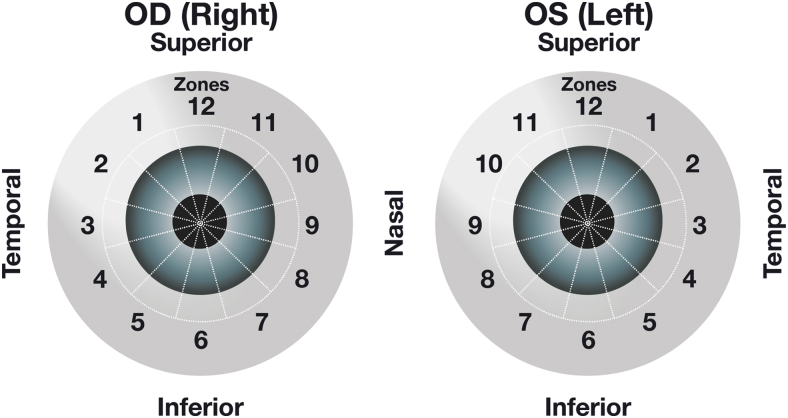
Zones of implant location within the iridocorneal angle. The location of each implant within 1 of 12 zones arranged circumferentially around the iridocorneal angle was determined during gonioscopic examination. OD, right eye; OS, left eye.

Implants were assessed on gonioscopy throughout the studies. The investigator reported whether implants were visible, identified the administration cycle for each visible implant (first, second, or third administration), and estimated the size of each visible implant compared with the initial implant size observed immediately after administration. Each investigator was given clear instructions on how to estimate the implant size, including use of the slit lamp beam as a reference for the size estimation. The investigator categorized and recorded the implant size as 0%–25%, 26%–50%, 51%–75%, 76%–100%, 101%–125%, 126%–150%, 151%–200%, or >200% of the initial size at implantation. The investigator also reported the location of each implant within iridocorneal angle zones, which were assigned around the corneoscleral limbus (Fig. 1).

Data for the 10-μg implant administered on day 1 (first administration) were pooled from both completed studies for analysis. The analysis of biodegradation included all patients with implant size assessment data at the earliest (week 2) follow-up study visit with gonioscopic examinations. The percentage of the implants administered on day 1 that were no longer visible, or that were estimated to be 0%–25%, 26%–50%, 51%–75%, 76%–100%, 101%–125%, 126%–150%, 151%–200%, or >200% of the initial size, was evaluated at follow-up visits through month 20 (study exit). Clinically significant implant biodegradation was defined as implant either absent or ≤25% of the initial size.

The percentage of visible implants located in each zone of the iridocorneal angle was evaluated at each follow-up visit through week 15 (the last visit before the second implant administration) using all observed data.

### Preclinical study

Studies of the pharmacokinetics of the bimatoprost implant in beagle dogs following a single intracameral administration were conducted at Covance Laboratories (Madison, WI) after approval of the study protocol by the Animal Care and Use Committee. The administration procedure used was described previously.^8^ Briefly, dogs were lightly sedated, then anesthetized, and study eyes were prepared for intraocular injection by irrigation with 5% povidone-iodine solution and placement of an eye speculum. The bimatoprost implant was injected into the anterior chamber using an applicator with a 25-gauge needle, which was inserted through the clear cornea in the superior temporal quadrant. An ophthalmic antibiotic ointment was applied in the cul-de-sac after the procedure.

After 3 to 6 months, the animals were euthanized with IV pentobarbital, eyes were enucleated, and ocular tissues and residual implant were collected as described previously.^9^ The retrieved implants were sent on dry ice to Allergan (now an AbbVie company) for analysis. Scanning electron microscopy (SEM) of implants before injection and the retrieved implants was performed for assessment of implant surface morphology and biodegradation characteristics. The implants were freeze-dried (LyoStar II lyophilizer; FTS Systems, Inc., Stone Ridge, NY) and prepared for SEM using a K550X Sputter Coater (Emitech Ltd., Kent, United Kingdom) to coat the implant surface with a thin (12 nm) layer of gold. SEM images were acquired with a Zeiss EVO 40 scanning electron microscope (Oberkochen, Germany) equipped with a secondary electron detector.

## Results

**FIG. 2.**
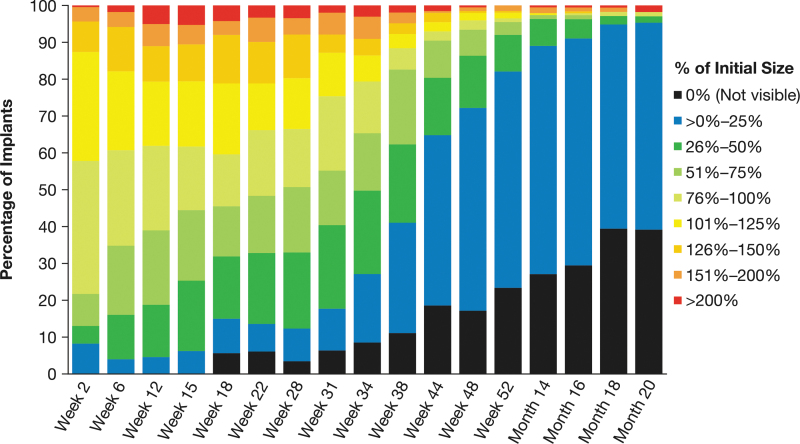
Distribution of implant size by time after administration. Patients were administered a 10-μg bimatoprost implant on day 1 in the ARTEMIS studies. During gonioscopic examination at follow-up visits, the size of the implant was estimated and categorized by percent initial size. Results shown are based on observed values (*n* = 230 at week 2).

A total of 372 patients received a 10-μg bimatoprost implant on day 1 of the ARTEMIS studies. Biodegradation of the implants was evaluated on gonioscopy and analyzed for all patients who had an implant size assessment at week 2 (*n* = 230). At each time point through month 20, implant size assessments showed variability in the degree of implant biodegradation among patients (Fig. 2).

**FIG. 3.**
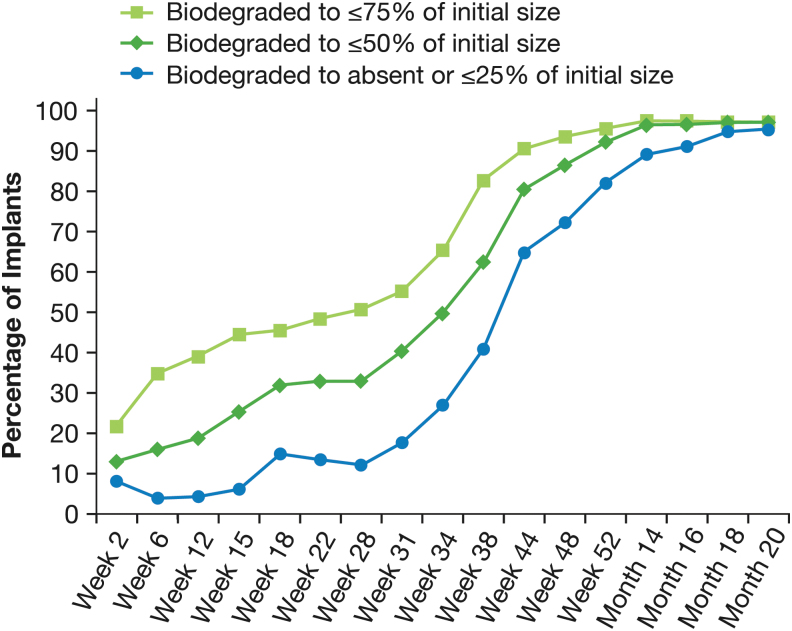
Time course of the biodegradation of the implant. Results shown are based on observed values (*n* = 230 at week 2).

During the initial phase of biodegradation from 2 to 28 weeks, implants were often reported to swell (Fig. 2). Typically, the implant size was reported to increase up to 50% over the initial size, with doubling of the size sometimes reported. At 12 weeks, 27.1% (59/218) of implants were estimated to be 101% to 150% of their initial size, and 11.0% (24/218) were estimated to be 151% to >200% of their initial size. The proportion of implants that biodegraded to ≤75% or ≤50% of their initial size increased during this initial phase (Fig. 3). At 28 weeks, 50.7% (103/203) of implants were estimated to be ≤75% of their initial size, and 33.0% (67/203) were estimated to be ≤50% of their initial size. Clinically significant biodegradation to absent or ≤25% of initial size was reported for 12.3% (25/203) of implants at 28 weeks.

Accelerated biodegradation occurred between 31 and 52 weeks, with most implants showing clinically significant biodegradation during this period. At week 52, 82.1% (165/201) of implants were reported to be absent or ≤25% of their initial size, 92.0% (185/201) were reported to be ≤50% of their initial size, and 95.5% (192/201) were reported to be ≤75% of their initial size (Fig. 3). After week 52, implants that remained visible continued to biodegrade. By month 20, 95.3% (163/171) of implants had biodegraded to absent or ≤25% of their initial size (Fig. 3).

**FIG. 4.**
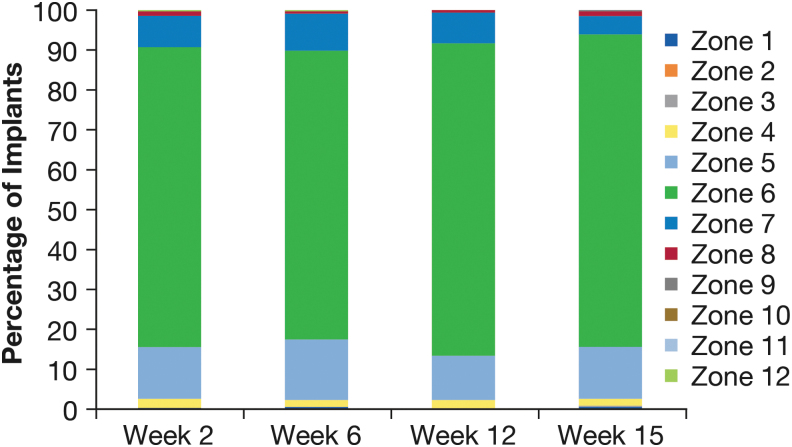
Implant location in the iridocorneal angle.

Gonioscopic examinations also included assessment of the implant location. The implant typically settled in the inferior iridocorneal angle. At each visit through week 15, ≥72.5% (258/356) of visible implants were located in Zone 6, inferiorly in the iridocorneal angle, and ≥96.0% (332/346) were located in Zone 5, 6, or 7 inferiorly (Fig. 4).

### Preclinical study

**FIG. 5.**
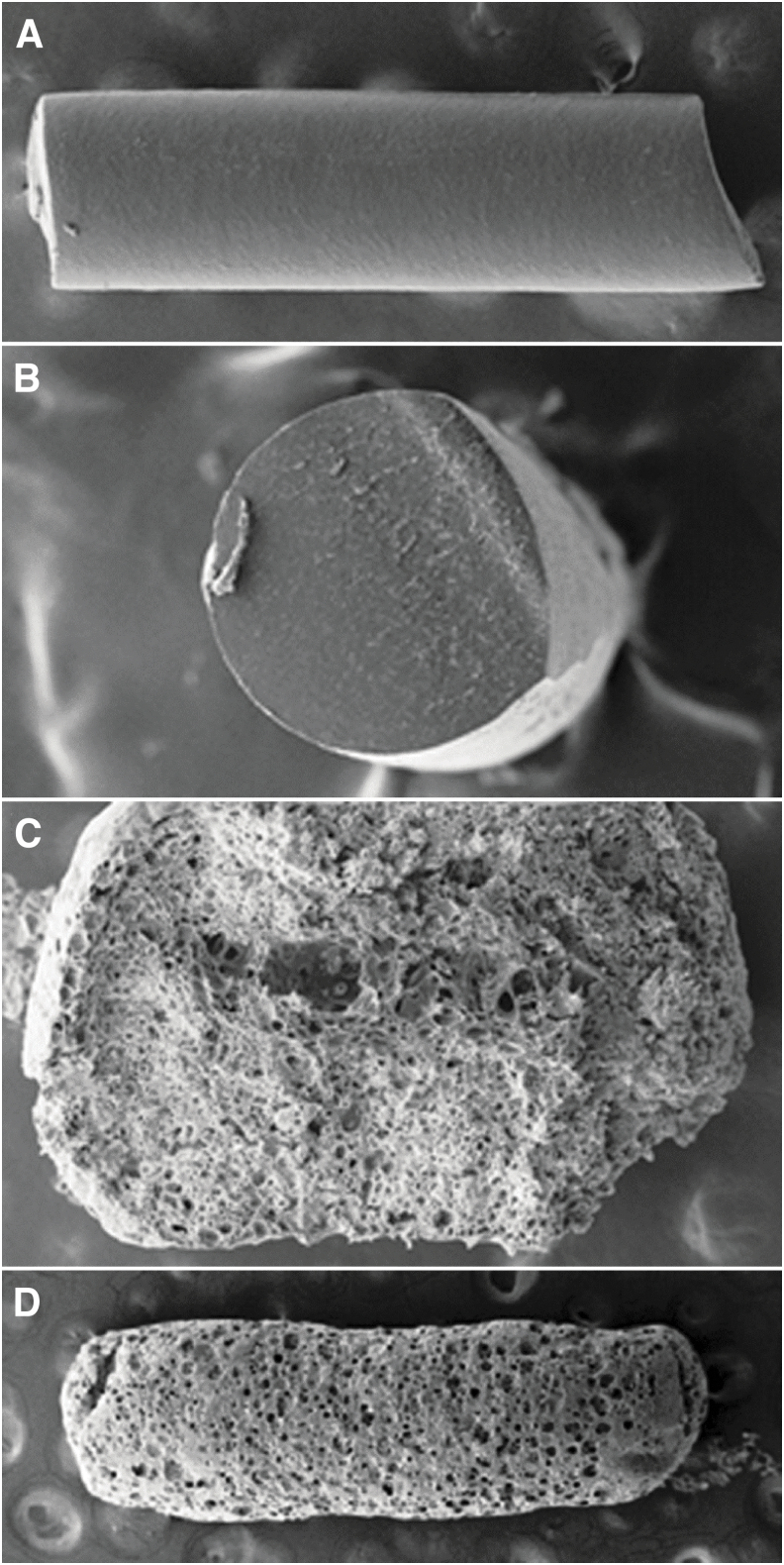
SEM images of representative bimatoprost implants before and 3–6 months after intracameral injection in beagle dogs. **(A)** Side view and **(B)** cross-sectional view of the rod-shaped bimatoprost implant before intracameral injection. **(C)** Cross-sectional view of an implant retrieved from a dog eye 3 months after injection. The implant material has become porous, indicating polymer biodegradation, and central coring is evident. **(D)** Side view of an implant retrieved from a dog eye 6 months after injection. The surface morphology demonstrates extensive pore formation and polymer biodegradation. SEM, scanning electron microscopy.

The surface morphology and biodegradation characteristics of the bimatoprost implant were evaluated with SEM in a previously unpublished preclinical study. In this study, each eye of 10 beagle dogs was injected intracamerally with a bimatoprost implant. The implants were retrieved after 3 to 6 months. Figure 5 shows representative photomicrographs of implants preinjection and postinjection. Before injection, the surface of the implants appeared smooth on SEM. Implants retrieved after 3 months showed a change in size, with a twofold to threefold increase in diameter, and a small decrease in length. The surface of the implants showed some pore formation, and the interior of the implants displayed porosity and central coring, indicating biodegradation. The surface of implants retrieved after 6 months showed extensive pores.

## Discussion

This study showed some variability in the rate of bimatoprost implant biodegradation among patients, for reasons that are unknown. Overall, there appeared to be 3 discrete stages of degradation of the implant administered on day 1 in the phase 3 ARTEMIS studies. During the first stage (approximately week 2 to 28), the majority of the implants remained at greater than 75% of their initial size. An increase in implant size to greater than 125% of the initial size was observed during this stage in some patients, most commonly from week 12 to week 28. This initial swelling was expected because of implant hydration when it contacts the aqueous.

**FIG. 6.**
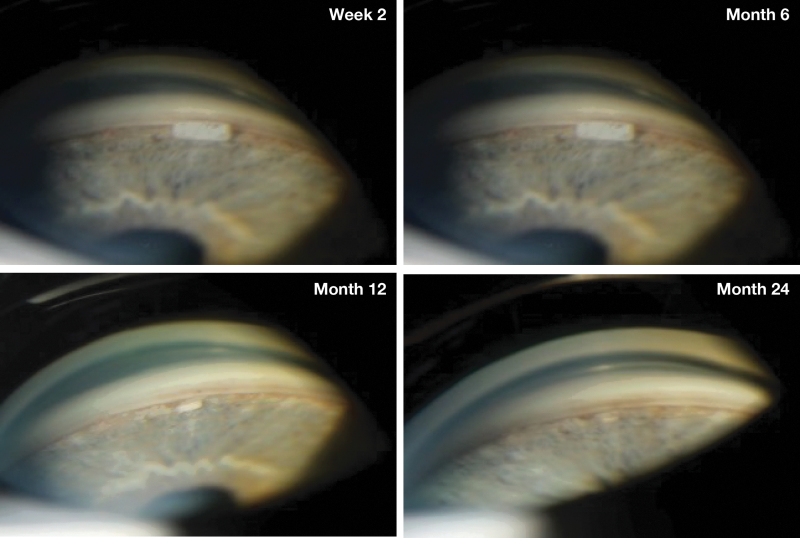
Gonioscopic photographs of a bimatoprost implant in the iridocorneal angle. The study eye of a patient with open-angle glaucoma was administered a single 10-μg bimatoprost implant in a phase 1/2 study.^3,4^ Gonioscopic photographs of the iridocorneal angle were taken at 2 weeks and 6, 12, and 24 months after the intracameral administration of the implant.

During the second stage (approximately week 31 to 52), a more rapid degradation occurred, with the majority of implants decreasing to less than or equal to 50% of their initial size by week 38, and to either no longer visible or 0% to 25% of their initial size by week 44. During the third stage (approximately week 52 to month 20), implant degradation continued at a slower rate, with more than 90% of implants either no longer visible or decreased to 0% to 25% of their initial size by month 20. The time course of implant biodegradation observed in the ARTEMIS studies was consistent with that seen in patients who received a single bimatoprost implant administration in an earlier phase 1/2 study (Fig. 6).

The implant resided in the inferior iridocorneal angle, at a clock hour of 5, 6, or 7, in the vast majority of gonioscopic evaluations, which were conducted with the patient sitting at the slit lamp. This location could be predicted from the influence of gravity on the implant. Potential movement of the implant associated with changes in head position and changes in the implant location between study visits were not evaluated.

During the early development of the bimatoprost implant, the preclinical study reported here evaluated the size and surface morphology of implants after intracameral administration in dogs. Implants retrieved after 3 months showed significant changes in size and surface porosity, and central coring was evident. The change in implant size is thought to be due to a combination of pore formation caused by polymer degradation and polymer chain relaxation. During the hot melt extrusion process used to disperse bimatoprost through the polymer matrix of the implant, the polymers were forced through a small nozzle. The resulting high shear force likely caused the polymer chains to be stretched and oriented along the axis of the implants. When the implant was injected intracamerally, water molecules, acting as plasticizers, diffused into the polymer matrix and facilitated the polymer chain relaxation. As a result, the diameter of the implant increased, and the length decreased.

The preclinical study further showed that implants had a porous structure after 3 months in the anterior chamber. The pores were more numerous and enlarged in the interior of the implant, suggesting that implant degradation and polymer erosion preferentially occurred from the inside out. Central coring may occur because degradation products of the polymers acidify the microenvironment within the implant,^10^ leading to a local acceleration in the biodegradation rate.

**FIG. 7.**
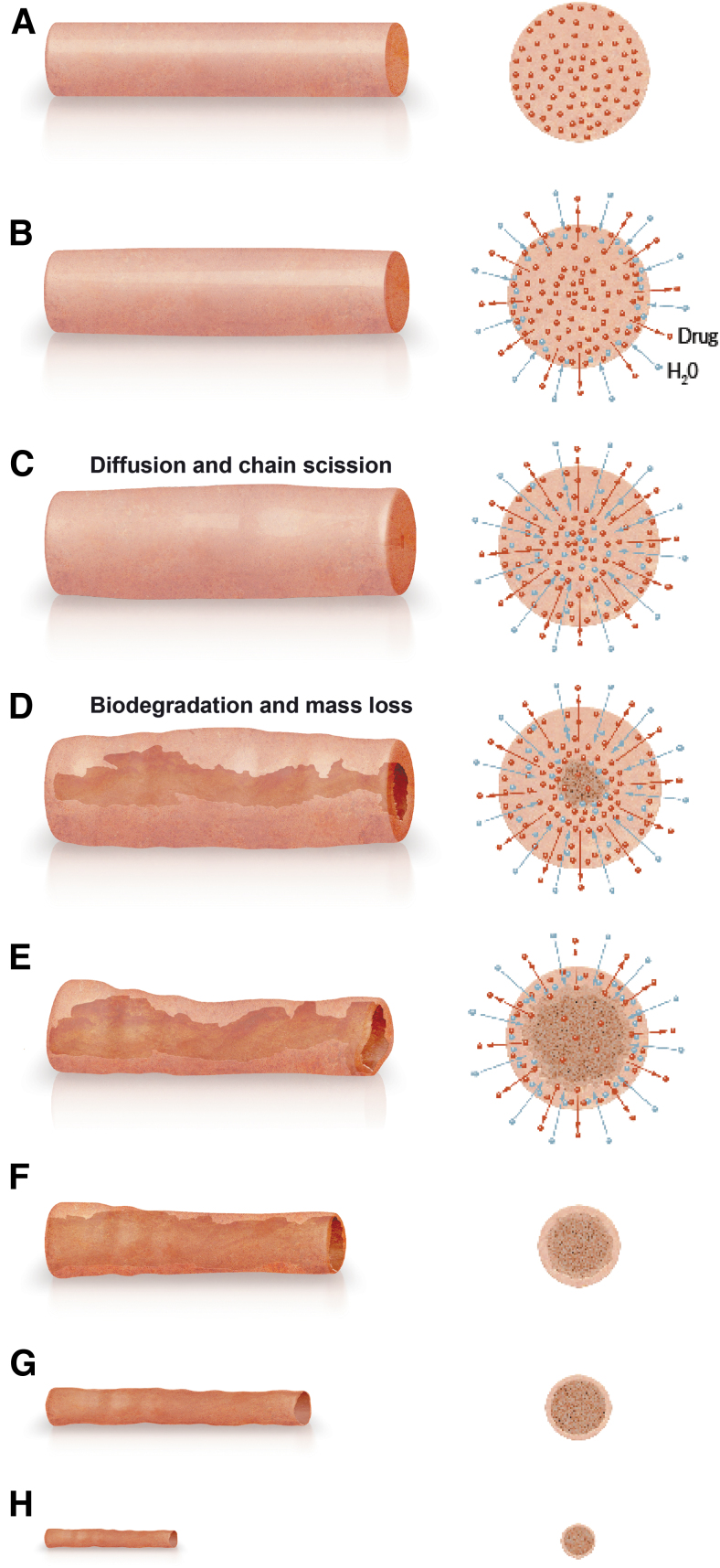
Schematic of bimatoprost implant biodegradation. Side view (*left*) and cross-sectional (*right*) images illustrate the initial implant **(A)** and the changes in implant size and structure that occur after the implant is placed in the anterior chamber **(B–H)**.

Figure 7 illustrates the biodegradation process after the bimatoprost implant is placed in the anterior chamber. Implant hydration begins immediately and is accompanied by drug release that continues in a linear manner until the implant is empty. Water molecules diffuse into the interior of the implant, resulting in polymer chain relaxation and scission, and implant swelling. Biodegradation with central coring occurs as the polymer chain scission produces low-molecular-weight polymers that erode from the implant. The biodegradation continues after the implant is emptied of drug. Central coring becomes more extensive as there is a steady erosion of the polymer mass, and the implant collapses, with a continuing reduction in size (both diameter and length) of the implant.

Because remnant implant may persist for months after complete drug release, the continued presence of the bimatoprost implant does not ensure drug release and associated IOP lowering. Conversely, IOP lowering can be maintained after the implant is completely biodegraded. This was documented in the phase 1/2 clinical study evaluating various dose strengths of the bimatoprost implant.^4^ Twenty-one participants completed the 24-month study without receiving rescue topical IOP-lowering medication or a second administration of the implant. At month 24, the mean IOP in the study eye of these participants was controlled at 16.9 mmHg, yet 28.6% of the participants (6 of 21) had no visible implant remaining in the study eye on gonioscopic examination.

The mechanism of IOP lowering by prostaglandin analogs, including bimatoprost, involves the upregulation of matrix metalloproteinases (MMPs) in the ciliary body and trabecular meshwork.^11–21^ The enhanced MMP activity results in increased extracellular matrix turnover and tissue remodeling, which decreases the resistance to aqueous outflow through the unconventional (uveoscleral) and conventional (trabecular meshwork) pathways and results in lowered IOP.

Drug-distribution studies in dogs have shown that the bimatoprost implant is more effective than topical dosing in delivering bimatoprost to target tissues, with high (micromolar) concentrations of bimatoprost achieved in iris–ciliary body samples after implant administration.^9^ As the effects of bimatoprost on MMP expression by ciliary muscle and trabecular meshwork cells are concentration dependent,^21^ we have proposed that the ability of the bimatoprost implant to provide sustained IOP lowering, even when the implant has completely degraded and tissue levels of drug are negligible, may be explained by the high levels of bimatoprost achieved in outflow tissues after bimatoprost implant administration producing enhanced upregulation of MMPs, which leads to more durable tissue remodeling and sustained IOP lowering.^1,4,8,21^

In clinical studies, there have been no signs of anterior segment inflammation attributable to the presence of residual implant material. Nevertheless, another potential explanation for the sustained IOP reduction over time could be the presence of residual implant material causing subclinical inflammation and release of endogenous prostaglandins. However, this theory does not explain the sustained IOP lowering observed in some patients who have no residual implant present in the eye.

This study analyzed the biodegradation of the first (day 1) implant received by participants in the ARTEMIS studies. One study limitation is the possibility that the administration of a second and third implant at week 16 and 32, respectively, could have affected the rate of biodegradation of the first implant. There is also a possibility that the first implant was not correctly identified in some cases when more than one implant was present. Finally, the investigators provided subjective estimates of implant size relative to the size of the initial implant, and the intersubject and intrasubject variability of the estimates is unknown.

Corneal adverse events occurred in the ARTEMIS studies when 10- or 15-μg bimatoprost implant was re-administered at 16-week intervals, with a higher incidence of these events associated with the larger 15-μg implant. Consequently, a single administration per eye of the smaller 10-μg bimatoprost implant is currently indicated to lower IOP in patients with open-angle glaucoma or ocular hypertension. However, the long duration of IOP lowering frequently observed after implant administration suggests the potential for safe and effective re-administration of the implant using a longer dosing interval. Ongoing, open-label clinical studies (NCT03850782, NCT03891446) are evaluating the safety and efficacy of as-needed administration of the implant.

## Conclusions

We have demonstrated that bimatoprost implant biodegradation in the ARTEMIS phase 3 studies showed some degree of variability among patients. Clinically significant implant biodegradation was observed in most patients by 12 months. Clinical studies are in progress to further understand implant biodegradation and the ideal timing for implant re-administration.

## Supplementary Material

Supplemental data

## Data Sharing Statement

AbbVie is committed to responsible data sharing regarding the clinical trials we sponsor. This includes access to anonymized, individual, and trial-level data (analysis data sets), as well as other information (eg, protocols, clinical study reports, or analysis plans), as long as the trials are not part of an ongoing or planned regulatory submission. This includes requests for clinical trial data for unlicensed products and indications.

The clinical trial data can be requested by qualified researchers who engage in rigorous, independent scientific research, and will be provided following review and approval of a research proposal and Statistical Analysis Plan (SAP) and execution of a Data Sharing Agreement (DSA). Data requests can be submitted at any time after approval in the US and Europe and after acceptance of this manuscript for publication. The data will be accessible for 12 months, with possible extensions considered. For more information on the process, or to submit a request, visit the following link: https://www.abbvieclinicaltrials.com/hcp/data-sharing/.
