# Supplementary material for: Bimatoprost Implant Biodegradation in the Phase 3, Randomized, 20-Month ARTEMIS Studies
Source: J Ocul Pharmacol Ther. 2023 Jan 25;39(1):55–62. doi: 10.1089/jop.2022.0137 (PMC9885540; doi:10.1089/jop.2022.0137)
Supplement: Supplemental data [file Suppl_FigS1.pdf]

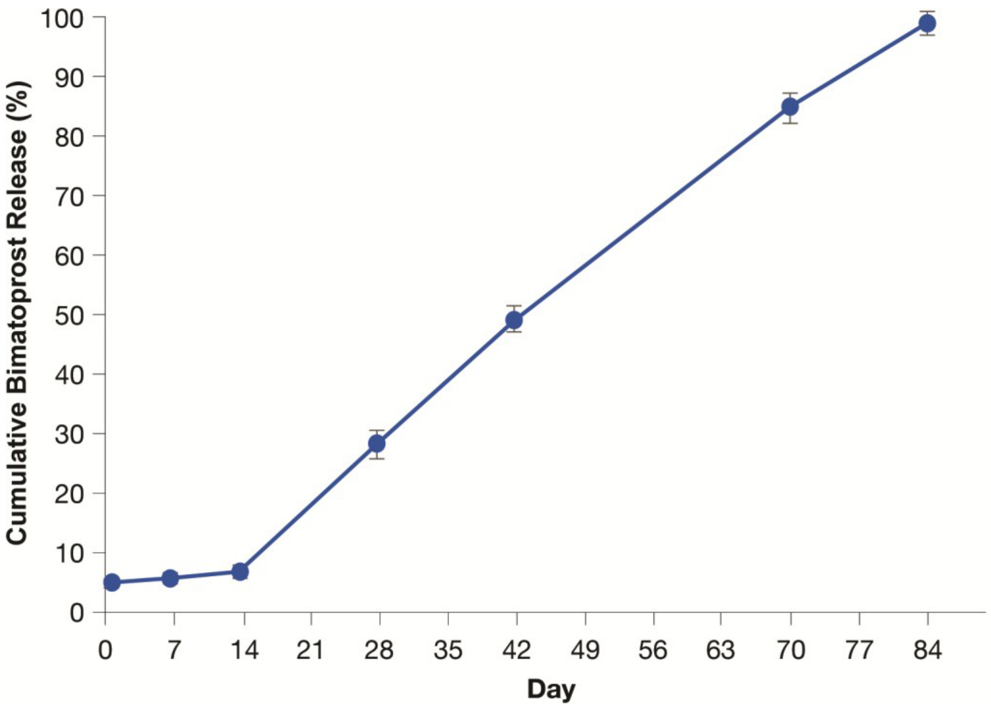

Supplemental Fig. S1. In vitro drug release profile of 10-μg bimatoprost implant over time. On day 0, implants were placed in 10 mM phosphate-buffered saline solution at pH 7.4 ± 0.1 and incubated at 32°C. Samples of the release media were obtained on days 1, 7, 14, 28, 42, 70, and 84; bimatoprost content of the samples was determined with a reversed phase high performance liquid chromatographic method. Cumulative percentage bimatoprost release was calculated based on the initial bimatoprost content of the implants (10 μg). Data shown are means ± standard deviation (n=6). Republished from Medeiros FA, et al. Phase 3, randomized, 20-month study of bimatoprost implant in open-angle glaucoma and ocular hypertension (ARTEMIS 1). *Ophthalmology.* 2020;127(12):1627–1641 under the terms of the Creative Commons CC-BY-NC-ND license.
